# Combination drug therapy against OAB normalizes micturition parameters and increases the release of nitric oxide during chemically induced cystitis

**DOI:** 10.1002/prp2.564

**Published:** 2020-02-07

**Authors:** Bhavik Patel, Fernando Perez, Patrik Aronsson, Ranya Alothmani, Thomas Carlsson, Michael Winder

**Affiliations:** ^1^ Department of Pharmacy and Biomolecular Sciences University of Brighton Brighton UK; ^2^ Centre for Stress and Age‐Related Diseases University of Brighton Brighton UK; ^3^ Department of Pharmacology Institute of Neuroscience and Physiology The Sahlgrenska Academy University of Gothenburg Gothenburg Sweden

**Keywords:** cyclophosphamide, cystitis, micturition parameters, mirabegron, nitric oxide, tolterodine, urinary bladder

## Abstract

Today, monotherapy is the most common pharmacological treatment option for patients suffering from overactive bladder (OAB). Recent reports have indicated potential benefits of combination therapy, using a muscarinic antagonist and a β_3_‐adrenoceptor agonist. This may be of particular interest for therapy‐resistant patients with OAB and concomitant cystitis. The objective of the current study was to assess how combination therapy affects bladder parameters in health and cystitis and if the efficacy of the drugs can be linked to altered release of nitric oxide (NO). Rats were pretreated with either a combination of the muscarinic antagonist tolterodine and β_3_‐selective adrenoceptor agonist mirabegron or saline for 10 days. Forty‐eight hours prior to assessing micturition parameters in a metabolic cage, the rats were intraperitoneally injected with cyclophosphamide, causing cystitis, or saline. Urine samples were collected and analyzed for NO content. Bladder contractile properties were assessed in an organ bath setup. Induction of cystitis led to bladder overactivity. Combination therapy normalized bladder parameters. Both induction of cystitis and drug treatment increased the release of NO. The innate contractile properties of the bladder were unaffected by combination therapy. This study demonstrates positive effects of combination drug therapy on symptoms of OAB, possibly indicating it to be a good option for treatment of OAB during concomitant cystitis. It remains to be determined if increased release of NO is crucial for successful pharmacological treatment of bladder overactivity during cystitis.

**Abbreviations**
CYP: Cyclophosphamide
DMSO: Dimethyl sulfoxide
DPV: Differential pulse voltammetry
IC: Interstitial cystitis
L‐NNA: N^ω^‐nitro‐L‐arginine
MeCh: Methacholine
NO: Nitric oxide
OAB: Overactive bladder
PB: Phosphate buffer
PBS: Phosphate‐buffered saline
PFA: Paraformaldehyde

## 1 INTRODUCTION

Overactive bladder (OAB) is a common medical condition with a prevalence of approximately 16%,^1^ which is characterized by urgency and frequency, often together with periods of nocturia and incontinence. The inflamed bladder has contractile properties that are somewhat similar to that of OAB, in particular during interstitial cystitis (IC), that is, bladder inflammation in the absence of bacterial infection. Namely, IC is apart from lower abdomen pain also commonly associated with frequency and urgency. However, even though there is sometimes an overlap in the pharmacological treatment of IC and OAB, there are also differences. While there are clear guidelines for the treatment of OAB, there is yet to emerge a worldwide consensus and effective pharmacological treatment option for IC.

A few years ago, mirabegron, an adrenoceptor agonist selective for the β_3_‐subtype, was approved for the treatment of OAB.^2^ At the time, the by far most common pharmacological therapy against OAB were antimuscarinic drugs. However, the clinical outcome of monotherapy with antimuscarinic drugs is often unsatisfactory and antimuscarinics commonly have side effects such as dry mouth, dry eyes, and constipation that often lead to discontinuation of the treatment, despite positive effects on the OAB symptoms.^3^ Leading up to its approval, both preclinical and clinical studies had shown beneficial effects of mirabegron against urgency and frequency,^4^, ^5^ with less reported side effects.^6^ In particular, it was shown to often be effective for the rather large group of patients who were unresponsive to antimuscarinic treatment.^7^ Today, monotherapy is still by far the most common treatment against OAB. For instance, patients in Europe and North America are usually first prescribed an antimuscarinic drug, and only if that does not alleviate the symptoms mirabegron is used. However, recent clinical studies have shown potential benefits of combination therapy with mirabegron and an antimuscarinic drug.^8^, ^9^, ^10^ This combination has therefore been suggested to have the potential of becoming the new golden standard treatment of OAB.

Part of the efficacy of mirabegron has been suggested to depend on induced release of nitric oxide (NO), tentatively from the urothelium.^11^ Even though it has been shown that β‐adrenoceptor activation causes the release of NO from human urothelial cells,^12^, ^13^ the actual effect of mirabegron on NO release in the bladder remains to be determined in conclusive studies. Considering previous findings that antimuscarinics could potentially decrease the release of NO, mainly in a state of cystitis,^14^, ^15^ and that this could be part of the reason for their lack of efficacy in a state of concomitant bladder overactivity and inflammation, the exact role that mirabegron has on NO release is highly interesting.

This study aims to assess the effects of treatment with combination drug therapy commonly used in the treatment of OAB. In particular, it is wondered if combination therapy leads to altered release of NO. It was also wondered how the combination therapy affects bladder parameters in health and cystitis. For this purpose, to obtain steady state, rats were pretreated with either saline or a combination of tolterodine (muscarinic antagonist) and mirabegron (β_3_‐selective adrenoceptor agonist) for 10 days. Forty‐eight hours prior to assessment of bladder parameters in a metabolic cage, rats from each group were given an intraperitoneal bolus dose of either saline, serving as control, or cyclophosphamide (CYP), a cytostatic drug commonly used to induce cystitis. During the metabolic cage assessment period, urine samples were collected and subsequently analyzed for NO content. After the assessment period, the rats were euthanized, the bladders were excised, and full‐thickness strips of bladder tissue were used for contractile studies in an organ bath setup.

## 2 MATERIAL AND METHODS

### 2.1 Animals

This study was approved by the local ethics committee at the University of Gothenburg (ethical permit 196‐13). A total of 32 male Sprague‐Dawley rats (285‐495 g, 10‐16 weeks) purchased from Charles River were used in this study. All experimental procedures followed the requirements stated in the ethical permit and had full adherence to ARRIVE and BJP guidelines. The species was chosen since it is phylogenetically close enough to man to allow for it to be translational and for the fact that most lower urinary tract animal models, including those previously performed by the authors, have been developed in rats. Extensive and relevant data therefore exist for valid comparisons to be made.

### 2.2 Study design

**Figure 1:**
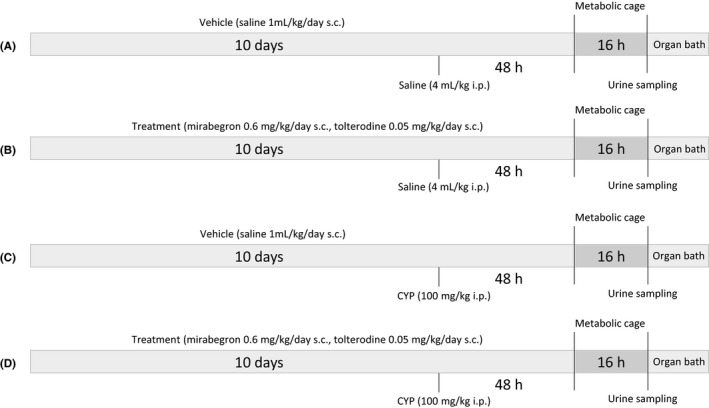
Experimental design. Rats were treated for 10 days with either vehicle (A, C; saline; 1 mL kg^−1^ per day *s.c.*) or a drug combination (B, D; mirabegron 0.6 mg kg^−1^ per day *s.c.* and tolterodine 0.05 mL kg^−1^ per day *s.c.*). Forty‐eight hours prior to spending 16 hour in a metabolic cage, the rats received an intraperitoneal injection with either saline (A, B; 4 mL kg^−1^) or cyclophosphamide (C, D; CYP; 100 mg kg^−1^) in order to induce cystitis

Each animal was randomized to be pretreated twice daily for 10 days with either saline (0.9% NaCl in 3% DMSO, 1 mL kg^−1^ per day *s.c.*, serving as control) or a combination of tolterodine (in saline; 0.05 mg kg^−1^ per day *s.c.*; 1 mL kg^−1^ per day) and mirabegron (in saline with 6% DMSO; 0.6 mg kg^−1^ per day *s.c.*; 1 mL kg^−1^ per day). The doses were chosen to match those recommended for the treatment of OAB patients and the time period was chosen to ensure reaching steady state of the drugs. Forty‐eight hours prior to assessment of bladder parameters in a metabolic cage, half of the animals in each group were randomized to be treated with a bolus dose of either saline (1 mL kg^−1^ *i.p*), serving as control, or the antineoplastic drug CYP (in saline; 100 mg kg^−1^ *i.p*,), in order to induce experimental cystitis (Figure 1). This generated four groups (saline pretreated controls, drug pretreated controls, saline pretreated inflamed, and drug pretreated inflamed) with eight animals in each group. After a 16‐h period in the metabolic cage, the animals were euthanized by an overdose of pentobarbitone (100‐150 mg kg^−1^ *i.p*; pentobarbital; APL), in accordance with the requirements stated in the ethical permit, and their bladders were excised for further assessment in an organ bath setup (n = 6).

### 2.3 Metabolic cage

After completion of the drug treatment protocol, micturition parameters were collected by placing the animals in a metabolic cage with free access to water. During a 16‐h observation period, urine samples from each rat were collected in a graded bottle and subsequently stored at −80°C and used for further (voltammetric) analysis. A WFL30‐40B416 laser Doppler (SICK) placed near the bottom of the metabolic cage continually registered each expelled drop of urine allowing for micturition parameters to be calculated. The number of micturitions was determined by visual analysis of the registered data. By division with the total time (16 hours), the number of micturitions per hour was calculated. To calculate the volume per micturition, the total volume was divided by the number of micturitions. Total water consumption was noted for each animal. Data were recorded using a MP150WSW data acquisition system and the AcqKnowledge 3.8.1 software (BioPac Systems).

### 2.4 Voltammetric measurement of nitrite

Urine samples stored at −80°C were shipped from the University of Gothenburg to the University of Brighton and analyzed in a blinded fashion. Upon analysis, samples were allowed to thaw on ice and then vortexed for 2 minutes prior to nitrite measurements. The experimental setup consisted of a 2‐mm platinum working electrode, an Ag|AgCl reference electrode, and a platinum wire counter electrode, used as a three electrode system (CH Instruments). Measurements were conducted using a 630b potentiostat with a faraday cage (CH Instruments). Data acquisition and analysis were achieved using the CH Instruments built‐in software.

For the measurement of nitrite standard and urine samples, differential pulse voltammetry (DPV) was utilized. The potential window was between 0.4 and 1.2 V, with a pulse width of 0.06 seconds and amplitude of 0.05 V. The scan rate was 0.004 V. Initially, DPV measurements were conducted using 0.5‐4 mmol L^−1^ sodium nitrite in 0.1 mol L^−1^ phosphate‐buffered saline (PBS) to generate a calibration response. Urine samples were then measured, with the electrode being polished between measurements. The current amplitude at the oxidation peak potential for nitrite was obtained from all the urine samples and converted to concentration by correlation to the calibration response. The measured levels of nitrite are generally considered to correspond to the levels of NO in urine.

### 2.5 Organ bath experiments

Most excised bladders were used for measurement of contractile properties. Upon removal of the bladder tissue, each specimen was visually examined for typical signs of inflammation, that is, redness and increased wall thickness. All bladders from animals pretreated with CYP displayed macroscopical signs of inflammation. Each bladder was cut along the opposite side of the trigone area from the urethral opening to the apex. The bladders were carefully pinned with the urothelial side up and full‐thickness bladder strips (6 × 2 mm) were prepared according to a standard procedure.^16^ Thereafter, the detrusor strips were mounted in 25‐mL organ baths containing Krebs solution (NaCl 118 mmol L^−1^, KCl 4.6 mmol L^−1^, KH_2_PO_4_ 1.15 mmol L^−1^, MgSO_4_ 1.15 mmol L^−1^, NaHCO_3_ 25 mmol L^−1^, glucose 5.5 mmol L^−1^, and CaCl_2_ 1.25 mmol L^−1^ in distilled water) at 37°C and gassed with 5% CO_2_ in O_2_ to maintain an appropriate pH level. The strips were stretched to 10 mN and let to equilibrate for 45 minutes. The baseline tension was continuously adjusted during the experiment to remain between 5 and 7 mN.

At the beginning of each experiment, a high K^+^ Krebs solution (KCl 124 mmol L^−1^, KH_2_PO_4_ 1.15 mmol L^−1^, MgSO_4_ 1.15 mmol L^−1^, NaHCO_3_ 25 mmol L^−1^, glucose 5.5 mmol L^−1^, and CaCl_2_ 1.25 mmol L^−1^ in distilled water) was administered in order to assess tissue viability. All drugs were administered to the organ baths at a volume of 125 μL. The muscarinic agonist methacholine (MeCh) was added cumulatively to the baths (10^−8^‐10^−4^ mmol L^−1^). After an initial MeCh dose‐response series, to assess possible functional influence of NO, N^ω^‐nitro‐L‐arginine (L‐NNA; 10^−4^ mmol L^−1^), an inhibitor of NO synthase, was added to the baths. After an incubation period of 20 minutes, the MeCh dose‐response series was run once more. At the end of the experiment, tissue viability was again tested by the addition of high K^+^ Krebs solution. Data were recorded using a MP36 data acquisition system and the AcqKnowledge 3.8.1 software (BioPac Systems).

### 2.6 Data analysis and statistical calculations

The data and statistical analysis comply with the recommendations on experimental design and analysis in pharmacology.^17^ Statistical analysis was undertaken only for studies where each group size was at least n = 5. The group sizes were calculated using the G*Power software ver 3.1.9.4.^18^ The calculation was based on an expected effect size of 2.5 (estimated from data from a previous study 13), *α* = .05, 1‐*β* = .95, and an allocation ratio of 1. To avoid underpowering, that is, to compensate for biological variation, a sample size of n = 8 was chosen for the current study. The declared group size is the number of independent observations and statistical analysis was done using these independent values. All values were included in the analysis and presentations, including possible outliers. Statistical significance was determined by two‐way analysis of variance (ANOVA) followed by Tukey's correction for multiple comparisons. Post hoc tests were conducted only if F in ANOVA achieved the level of statistical significance. Statistical significance was regarded for *p*‐values <.05. Data are presented as mean ± SEM. Graphs were generated and parameters computed using GraphPad Prism 8.1 (GraphPad Software Inc).

### 2.7 Materials

All substances and materials were purchased from Sigma‐Aldrich unless otherwise stated.

Tolterodine tartrate (Tocris), mirabegron (AdooQ Bioscience), cyclophosphamide monohydrate, pentobarbital sodium (pentobarbitone; APL, Gothenburg, Sweden), dobutamine hydrochloride (Tocris), acetyl‐β‐methylcholine chloride, N^ω^‐nitro‐L‐arginine (L‐NNA), sodium chloride, potassium chloride, potassium dihydrogen phosphate, magnesium sulfate, sodium bicarbonate, D‐(+)‐glucose, calcium chloride dehydrate, and dimethyl sulfoxide (DMSO).

## 3 RESULTS

### 3.1 Effects of drug treatment and cystitis on bladder parameters

**Figure 2:**
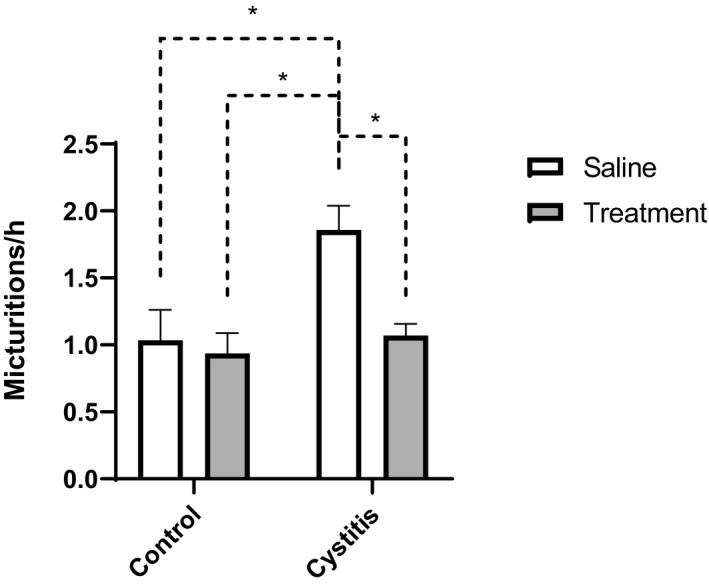
Number of micturitions per hour (frequency) in healthy (control) rats and rats treated with CYP in order to induce cystitis. In both groups, animals were pretreated for 10 days with either saline (□; 1 mL kg^−1^ per day *s.c.*) or a combination drug treatment (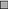) against OAB consisting of the muscarinic antagonist tolterodine (0.05 mL kg^−1^ per day *s.c.*) and the β_3_‐adrenoceptor agonist mirabegron (0.6 mL kg^−1^ per day *s.c.*). Induction of cystitis caused an increase in frequency, which could be normalized by OAB drug treatment. Statistical comparisons are made by two‐way ANOVA followed by Tukey's correction for multiple comparisons. n = 8 in all groups. *P < .05

In comparison to controls, induction of cystitis with CYP caused a significant increase in micturition frequency (1.03 ± 0.23 and 1.86 ± 0.18 mict h^−1^ in saline pretreated controls and saline pretreated inflamed, respectively; Figure 2). Combination drug therapy with tolterodine and mirabegron normalized the number of micturitions per hour (1.86 ± 0.18 and 1.07 ± 0.09 in saline pretreated inflamed and drug pretreated inflamed, respectively; Figure 2). Meanwhile, micturition frequency was not affected by combination drug treatment in healthy rats (1.03 ± 0.23 and 0.94 ± 0.15 mict h^−1^ in saline pretreated and drug pretreated control rats, respectively; Figure 2).

**Figure 3:**
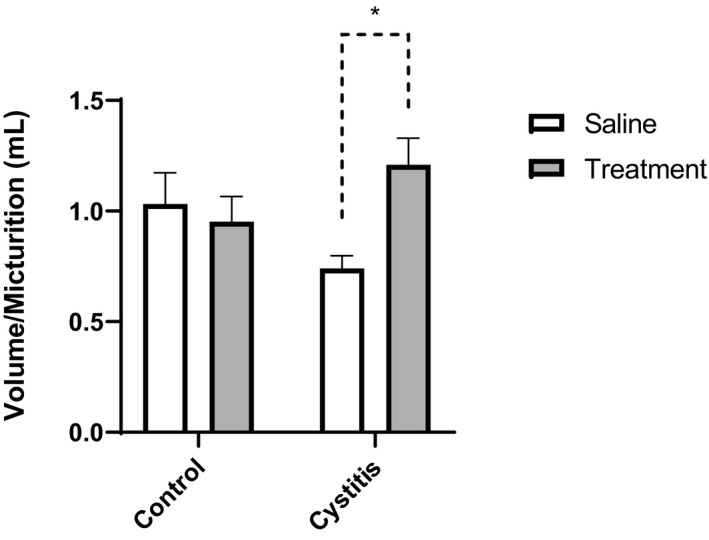
Volume per micturition in healthy (control) rats and rats treated with CYP in order to induce cystitis. In both groups, animals were pretreated for 10 days with either saline (□; 1 mL kg^−1^ per day *s.c.*) or a combination drug treatment (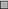) against OAB consisting of the muscarinic antagonist tolterodine (0.05 mL kg^−1^ per day *s.c.*) and the β_3_‐adrenoceptor agonist mirabegron (0.6 mL kg^−1^ per day *s.c.*). Induction of cystitis caused a decrease in micturition volume, which was normalized by OAB drug treatment. Statistical comparisons were made by two‐way ANOVA followed by Tukey's correction for multiple comparisons. n = 8 in all groups. *P < .05

Induction of cystitis with CYP caused a substantial but nonsignificant decrease of the volume per micturition (1.03 ± 0.14 and 0.74 ± 0.06 mL in saline pretreated controls and saline pretreated inflamed, respectively; Figure 3). In inflamed rats, combination therapy led to a significant increase in the volume per micturition (0.74 ± 0.06 and 1.21 ± 0.12 mL in saline pretreated inflamed and drug pretreated inflamed, respectively; Figure 3) to a level similar to that observed in controls. A similar increase in the micturition volume could not be observed in controls upon drug treatment (1.03 ± 0.14 and 0.95 ± 0.11 mL in saline pretreated and drug pretreated control rats, respectively; Figure 3).

### 3.2 Effects of drug treatment and cystitis on water consumption and total urine production

No significant differences could be detected between any of the groups with regard to water consumption neither due to drug treatment nor induction of cystitis, even though there was a tendency that CYP‐treated animals drank more (Table 1). However, the interindividual variations in water consumption were high among all groups. A similar tendency of an increased total volume of micturition can be seen in the CYP‐treated groups. This is likely due to the somewhat higher water intake in these groups.

**Table 1:** Total volume of micturition and water consumption in healthy (control) rats and rats treated with CYP in order to induce cystitis

	Control ‐ saline	Control ‐ treatment	Inflamed ‐ saline	Inflamed ‐ treatment
Total volume of micturition (mL per 16 h ± SD)	15.5 ± 8.6	14.6 ± 6.0	21.8 ± 11.0	23.0 ± 14.1
Water consumption (mL per 16 h ± SD)	16.6 ± 10.5	14.0 ± 9.7	25.2 ± 17.8	23.1 ± 15.4

Note

In both groups, animals were pretreated for 10 days with either saline (1 mL kg^−1^ per day *s.c.*) or a combination drug treatment against OAB consisting of the muscarinic antagonist tolterodine (0.05 mL kg^−1^ per day *s.c.*) and the β_3_‐adrenoceptor agonist mirabegron (0.6 mL kg^−1^ per day *s.c.*). No significant differences could be detected by statistical comparisons using two‐way ANOVA. n = 8 in all groups.

### 3.3 Effects of drug treatment and cystitis on the levels of nitric oxide

**Figure 4:**
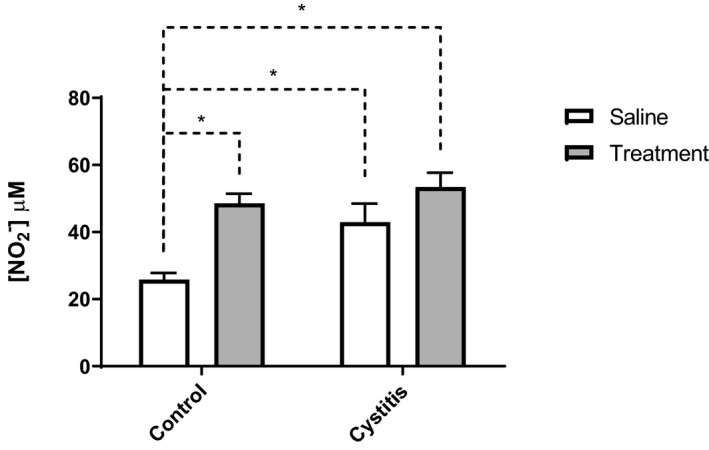
Levels of nitrite (corresponding to levels of NO) in urine from healthy (control) rats and rats treated with CYP in order to induce cystitis. In both groups, animals were pretreated for 10 days with either saline (□; 1 mL kg^−1^ per day *s.c.*) or a combination drug treatment (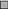) against OAB consisting of the muscarinic antagonist tolterodine (0.05 mL kg^−1^ per day *s.c.*) and the β_3_‐adrenoceptor agonist mirabegron (0.6 mL kg^−1^ per day *s.c.*). Both combination drug treatment and cystitis per se caused a significant increase in the levels of NO. Measurements were conducted by voltammetry and concentration of nitrite corresponds to levels of NO in urine. Statistical comparisons are made by two‐way ANOVA followed by Tukey's correction for multiple comparisons. n = 8 in all groups. *P < .05

Measured by voltammetry as amount of nitrite in urine, treatment with the combination therapy caused an increased release of NO in the urinary bladder both in healthy and inflamed rats (25.86 ± 1.96, 48.54 ± 2.88, and 53.42 ± 4.28 µmol L^−1^ in saline pretreated controls, drug pretreated controls, and drug pretreated inflamed, respectively; Figure 4). Further, induction of bladder inflammation per se increased the release of NO (25.86 ± 1.96 and 42.99 ± 5.51 µmol L^−1^ in saline pretreated controls and saline pretreated inflamed, respectively; Figure 4).

### 3.4 Effects of drug treatment and cystitis on detrusor contractility

**Figure 5:**
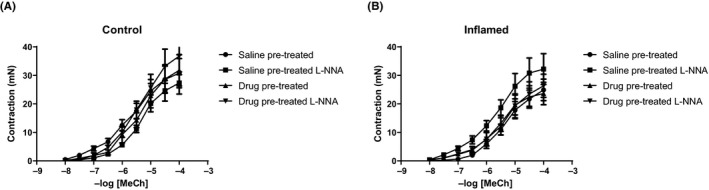
Mean contractile responses to methacholine in rat detrusor. Contractile responses in strips from (A) control bladders tended to be slightly higher than in strips from (B) bladders with CYP‐induced cystitis. No significant contractile differences could be seen in either control or inflamed strips when comparing responses between strips from saline and drug pretreated animals. Presence of the NO synthase blocker L‐NNA did not significantly alter the contraction‐response curves. Error bars represent ± SEM. n = 6 in all groups

In an organ bath setup, no significant differences in MeCh‐induced contraction could be detected between bladder strips from saline or drug pretreated rats, neither in control nor inflamed tissue (Figure 5). Likewise, addition of the NO synthase blocker L‐NNA did not significantly shift the contraction‐response curve, even though there was a tendency for an increase in the saline pretreated inflamed group (Figure 5B). In general, MeCh‐induced contraction was slightly greater in healthy (control) strips than inflamed bladder strips (Figure 5A and B, respectively).

## 4 DISCUSSION AND CONCLUSIONS

In the current study, induction of cystitis with CYP led to changes in micturition parameters causing a state of bladder overactivity. This is in line with previous studies.^19^, ^20^ Combination therapy with mirabegron and tolterodine normalized the altered micturition parameters, without affecting the micturition pattern in healthy rats. Simultaneously, the drug treatment did not significantly affect water intake or total volume of produced urine over a period of 16 hours, even though CYP‐treated rats tended to drink more and therefore produce a larger total volume of urine than controls. However, even though a somewhat larger water consumption could contribute to a change in the urodynamics, the overall changes of the micturition parameters are evident and agree well with previously reported changes occurring in CYP‐pretreated rats.

Positive effects of combination therapy have been shown in a previous preclinical study, which examined the acute effects of treatment with mirabegron and antimuscarinics on cystometric parameters in conscious female rats with bladder overactivity induced by oxotremorine methiodide.^21^ That study concluded that combination therapy significantly improved cystometric parameters as compared to monotherapy with either drug alone. Superior clinical outcome of combination therapy as compared to monotherapy has also been shown in a clinical study on OAB patients.^9^ The current study demonstrates similar positive effects of combination therapy on symptoms of bladder overactivity in a state of concomitant cystitis. However, it needs to be pointed out that CYP‐induced cystitis is a model of acute inflammation, different from chronic forms of bladder inflammation seen in patients, most commonly IC.^22^

While studies on the relaxatory effects of mirabegron in human and rat corpus cavernosum or human and rabbit prostate could not show any mechanistic involvement of NO,^23^, ^24^ others have suggested that NO is part of the relaxatory mechanism of mirabegron in the bladder.^11^ The current study shows that combination treatment causes release of NO in the urinary bladder. It is unlikely that this increased release is due to the action of tolterodine as previous studies have shown that, if anything, antagonism of muscarinic receptors attenuates NO release, in particular, in a state of inflammation.^14^ Thus, it is likely that mirabegron causes the release of NO. Further, the data show that induction of cystitis per se leads to an increased release of NO. This is expected as NO is commonly involved in inflammatory processes and NO previously has been attributed to an increased role in the urinary bladder during CYP‐induced cystitis.^14^, ^25^ However, it is not possible to detect a significantly increased release of NO in a state of cystitis during combination therapy as compared to saline treated rats with CYP‐induced cystitis. Considering that the micturition parameters are normalized by combination therapy, this could indicate that the increased release of NO during cystitis is not crucial to the mechanism of action of, mainly, mirabegron. However, several other possible explanations exist. For one, it is possible that the production capacity is maximized in a state of inflammation, rendering it impossible for mirabegron to further increase the release of NO. Another possible explanation is that the inflammatory response upon treatment with CYP is attenuated by the present combination therapy. In such a scenario, the measured release of NO in the inflamed bladders from rats under combination therapy would mainly be attributable to mirabegron. A third possible explanation is that inflammation per se is increasing the release of NO, but that the drugs included in the combination therapy are balancing each other out with regard to NO release. Namely, that tolterodine is decreasing but mirabegron is increasing the release of NO. This would be in line with the findings in previous studies.^14^, ^26^ However, for conclusive evidence, this would need to be examined in animals treated with only mirabegron or tolterodine, respectively.

The unchanged responses of isolated full‐thickness bladder strips to methacholine in an organ bath setup demonstrate that treatment with a combination of tolterodine and mirabegron does not significantly alter the innate contractile properties of the detrusor, at least not over a period of 10 days. This may seem surprising when considering that another study in rat has demonstrated an increased bladder sensitivity to muscarinic agonists as well as an increased expression of muscarinic receptors upon induction of overactivity.^27^ However, in that study, overactivity was induced by treatment with streptozotocin, which induces a diabetic state in the bladder. Preliminary experiments do not indicate altered expression of muscarinic receptors in the current study (data not shown). However, future studies should address this issue quantitatively. For obvious reasons, alterations in receptor expression and innate contractility over longer periods of combination drug therapy or in combination with other methods of induction of bladder overactivity cannot be excluded.

In accordance with previous studies, induction of inflammation with CYP attenuates the contractile cholinergic response of rat detrusor.^25^ Further, as previously shown,^28^ induction of cystitis with CYP gives rise to certain histological changes, that is, thickening of the bladder wall due to swelling and urothelial proliferation. This was obvious in the present study as well when studying the tissue under a light microscope (data not shown). Interestingly, proliferation of the urothelium was not as prominent in inflamed tissues from rats that underwent combination therapy. Notably, mirabegron has previously been shown to have protective effects in a rat model for chronic ischemic bladder.^29^ However, in order to draw more certain conclusions regarding the potential protective effects of mirabegron, this needs to be studied quantitatively in animals undergoing monotherapy.

In order to ensure correct blood concentration of tolterodine and mirabegron at the time of measurement of micturition parameters, it was imminent that the rats were pretreated with the drugs for a substantial time period. This inevitably led to the drugs being present at the time of induction of cystitis. Therefore, the possibility that the combination therapy had a dampening effect on the development of cystitis cannot be disregarded. Likewise, we cannot ignore the possibility of this affecting micturition parameters and NO release differently in a clinical situation in which cystitis typically develops before drug treatment is initiated.

In conclusion, combination therapy with mirabegron and tolterodine normalized altered micturition parameters that arose upon induction of inflammation. This was achieved without affecting the innate contractile properties of the bladder. Further, combination therapy led to an increased release of NO. It remains to be investigated if this increased release is a determining factor for the successful pharmacological treatment of symptoms of bladder overactivity. With a cautious approach to suggesting clinical implications, and without taking potential side effects into account, the findings in this study indicate that combination therapy may be the best treatment option for OAB patients with concomitant cystitis.

## DISCLOSURE

None of the authors have any conflict to declare.

## AUTHOR CONTRIBUTIONS

Study design: MW. Collection of data: MW, BP, FP, RA. Analysis, management and interpretation of data: MW, BP, FP, TC, PA. Preparation of manuscript: MW, BP, PA, TC, RA.

## DECLARATION OF TRANSPARENCY AND SCIENTIFIC RIGOR

This Declaration acknowledges that this paper adheres to the principles for transparent reporting and scientific rigor of preclinical research recommended by funding agencies, publishers, and other organizations engaged with supporting research.
